# DEGRADATION OF PARENTAL BONDING AND VIOLENCE AGAINST CHILDREN: THE USE OF FAMILY GENOGRAM IN THE PEDIATRIC CLINIC

**DOI:** 10.1590/1984-0462/;2017;35;2;00009

**Published:** 2017-05-15

**Authors:** Égle Thomaz Leoncio, Sonia Regina Pereira de Souza, José Lúcio Martins Machado

**Affiliations:** a Universidade Cidade de São Paulo, São Paulo, SP, Brasil.; b Faculdade de Medicina São Caetano do Sul, São Caetano do Sul, SP, Brasil.

**Keywords:** health vulnerability, children, family, genogram, qualitative research

## Abstract

**Objective:**

To demonstrate the importance of using the family genogram in pediatric consultation, as an analysis tool to evaluate the degradation of parental bonding and also violence against children.

**Methods:**

A qualitative study was conducted in 2011 wherein 63 children, aged between 2 and 6 years, enrolled in a slum nursery, was studied. In order to construct the genogram, data were collected in four stages: pediatric evaluation at nursery; interview with caregivers; interview with teachers; and interview with the nursery coordinator. The data about the families were used to construct the genograms with the aid of GenoPro^®^-2016 software. In order to evaluate the quality of bonding, the following items were included in the genograms: violence against children, drug addiction, neglect, mental disorder, type of relationship among family members.

**Results:**

The evaluated children and their families generated 55 genograms. In 38 of them, functional family arrangements, and close or very close emotional ties were observed. In 17 cases, situations involving physical, emotional, or sexual violence against children were perceived. Among these, four represented extreme cases, with fraying parental bonding, and dysfunctional family arrangements. In these families, chemical addiction was prevalent among multiple members, as well as severe mental disorder, persistent physical and verbal abuse, and sexual abuse.

**Conclusions:**

The use of the genogram helps to identify at an early stage the degradation of parental bonding and violence against children, and when it is incorporated into the pediatric practice routine, it may contribute to the promotion of the comprehensive health care of the child, regardless of the presence of social vulnerability.

## RESUMO

**Objetivo:**

Demonstrar a importância da utilização do genograma familiar na consulta pediátrica como ferramenta de análise da presença de degradação do vínculo parental e da violência contra a criança.

**Métodos:**

Estudo qualitativo, conduzido em 2011. A população compreendeu 63 crianças, com idades entre 2 e 6 anos, matriculadas em creche. Para a construção do genograma, a coleta dos dados se deu em quatro momentos: avaliação pediátrica na creche; entrevista dos cuidadores; entrevista dos professores; e entrevista da coordenadora da creche. Foram utilizados os dados sobre as famílias para a construção dos genogramas, com auxilio do programa GenoPro^®^-2016. Na avaliação da qualidade de vínculo, foram incluídos na representação do genograma: violência contra a criança, dependência química, negligência, transtorno mental, tipo de relação entre os membros da família.

**Resultados:**

As crianças e respectivas famílias avaliadas deram origem a 55 genogramas. Em 38 deles, observaram-se arranjos familiares funcionais e com vínculos afetivos próximos ou muito próximos. Em 17 dos casos, evidenciaram-se situações que envolviam violência física, emocional ou sexual contra as crianças. Dentre esses, quatro representavam casos mais extremos, com esgarçamento do vinculo parental e arranjos familiares disfuncionais. Nessas famílias predominava a dependência química de múltiplos membros, transtorno mental grave, agressões física e verbal persistentes e abuso sexual.

**Conclusões:**

A utilização do genograma auxilia na identificação precoce da degradação do vínculo parental e da violência praticada contra a criança e, quando incorporada à prática pediátrica rotineira, pode contribuir para a promoção de uma assistência integral à saúde da criança, independentemente da presença de vulnerabilidades social.

## INTRODUCTION

In modern society, a family arrangement is one of the different configurations that the family can embrace, such as multigenerational family, extended family, and reconstructed family.^1^ The motives that guide the lifestyle of a family depend on how the power relations are established, which determine family roles, its organization and performance.^2^ According to Kalostian and Ferrari in 1994, the family is an indispensable space for the insurance of survival and full protection of the children and other relatives.^3^ On the other hand, in a family in which misery, hunger, and violence are present, the household represents a place of privation, instability, and thinning of the affectionate and solidarity bonds, characterizing a situation of social vulnerability.^4^

The family can be both protective against the development of mental disorder and it can turn into a triggering factor. When the mother is an alcohol consumer, an environment with low cohesion and organization, as well as great incidence of domestic violence, is observed.^5^ Children who live with alcoholic mothers are exposed to negligence, abuse, and behavioral issues.^6^

Sierra and Mesquita analyzed children and adolescents and concluded that intra-household violence perpetrated by the parents, as well as privation and negligence, represent high chances of causing psychological and physical damage, and the consequent delay in the full development of the child.^7^ Another study, conducted by Brito and collaborators, showed that aggressive behavior and victimization that are present among adolescents, have been associated with physical domestic violence.^8^ In this context, specialized child care, without showing the complexity of family dynamics, is like caring for the symptom without treating the cause. The search for full care to the child indicates a new vision of health care and a huge challenge to clinical practice.^9^

Among the innovative practices that provide a broader view of the child’s universe, the family genogram stands out.^10^ The genogram is a visual-symbolic representation instrument that provides qualitative information about the dimension of family dynamics and functioning, demonstrating and organizing the genetic, medical, social, behavioral, relational, and cultural aspects involved in the family structure. Major events such as deaths, divorces, accidents, mental disorders, violence, chemical and alcohol dependence, and sexual abuse are also shown in this graphic representation, in order to plan for the full family health care.^11^ ^,^ ^12^ The familiarization of the pediatrician with this instrument increases the resoluteness of the service provided for the child, and it can also help the family to understand its members in new ways, as well as promote means to become partners in its own therapeutic plan.^13^

Therefore, this study aimed at describing the use of the family genogram in the pediatric appointment as a tool to analyze the degradation of parental bonding and violence against children.

## METHOD

The study consisted of a qualitative study, conducted in a philanthropic day-care facility in the city of Guarulhos, São Paulo. The institution is managed by a religious order and is located at the border of the road Fernão Dias. The population of this investigation included 63 children, aged between 2 and 6 years, enrolled in the day-care facility in 2011, corresponding to 55 family genograms.

To build the genogram, data collection about the children took place in four moments: at the pediatric evaluation in the daycare, by analyzing the school records; interviewing the person in charge of the child; interviewing the teachers; and interviewing the day-care coordinator. First, the mother or tutor was interviewed with a structured questionnaire. Then, the teacher in charge of the child was interviewed in order to obtain additional information such as parental involvement in the children’s routine at the daycare, health care, and signs of violence or sexual abuse. At last, the religious woman in charge of the facility was interviewed, since she had major influence on the community and had extensive knowledge about the families. In this interview, it was possible to collect information regarding the use of alcohol and other drugs by the family members, the quality and type of family bonds, and the history of physical and sexual abuse.

The structured questionnaire, applied during the interview of the mothers or tutors, involved the following data: general conditions of the child, identification and obstetric history of the mother, lifestyle and household, child’s medical history, family structure, qualification of the head of the family, work status, type of household, family monthly income, registration in the social program Bolsa Família, marital status, number of residents in the household, basic sanitation conditions, and presence of an electric network. Data about the families were used to build the family genograms, with the software GenoPro^®^-2016 (GenoPro, Waterloo, Ontario, Canada), in which the symbols represent the individuals, and the lines translate relationships between the individuals.^10^ ^,^ ^11^ In order to assess the quality of the relationship, the genogram representation included violence against children, presence of alcohol and/or drugs, negligence, and mental disorders.^12^ In the analysis of family genograms, parental bonding was rated from “very close” to “hate”. Regarding violence against children, the spectrum of analysis ranged from negligence to sexual abuse.

The research project was approved by the Research and Ethics Committee (CAAE n. 0051.1.186.000-11), and the Informed Consent Form was signed by the children’s parents or tutors after agreeing to participate in the study.

## RESULTS

The children assessed and their respective families generated 55 genograms; 38 showed functional family arrangements, with close or very close affectional bonds. Despite being socially vulnerable, most families had in place protective nuclear arrangements and functional dynamics for the child. By considering the levels in the spectrum of violence, in these families the mild form stood out. Parental bonding was strong, and physical or verbal aggression was a reproduction of models coming from paternal education. Another experience presented in these groups, with less frequency, was negligence. Parental bonding was weakened by paternal mental disorders, single-parent relationships, chemical dependence, or a mother with multiple partners.

**Figure 1:**
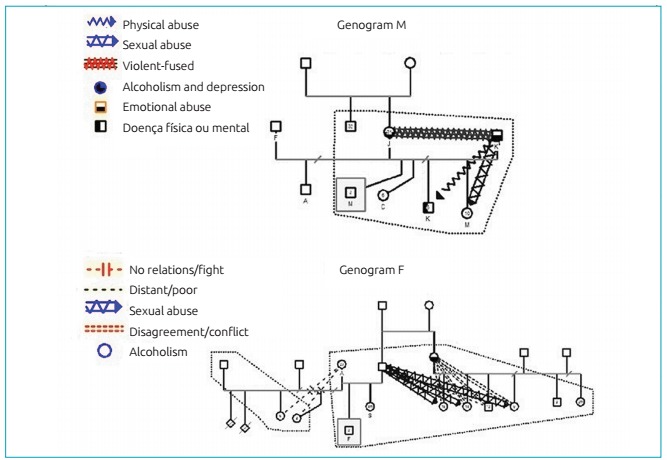
Genograms of M’s and F’s families.

**Figure 2:**
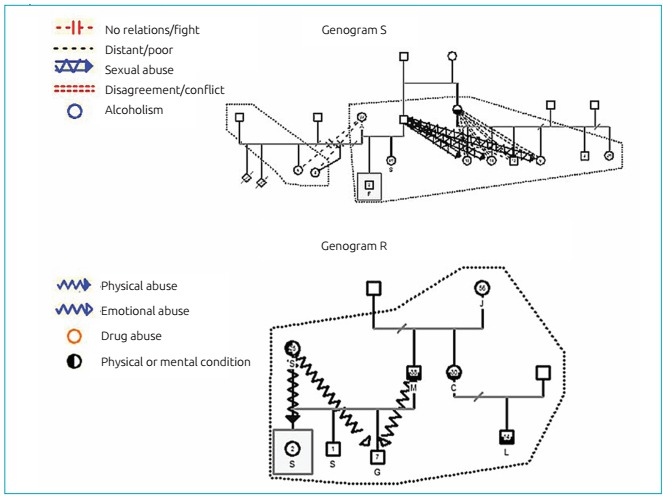
Genograms of S and R’s families.

In 17 of the analyzed cases, there were situations involving physical, emotional, or sexual violence against children. The most extreme cases of violence, with weak parental bonding, occurred in families with dysfunctional arrangements. In these cases, the prevalent scenario was chemical dependence of many family members, severe mental disorder, persistent verbal and physical aggression, and sexual abuse. The child often took the role of the adult, being in charge of taking care of family members. The results present the family genograms of children who have been victims of extreme violence. The child is represented by the first letter of his or her name (as shown in Figures 1 and 2).

Figure 1 (genogram M) shows that even though M’s family has a nuclear arrangement (father, mother, and children), its dynamic is dysfunctional, associated with the low quality of parental bonding. The paternal bond is extremely negative, presenting physical and sexual abuse in the relationship with the daughters, and violence-fused with the partner. In this family arrangement, the maternal bond is compromised by the presence of alcoholism, depression, overload in taking care of a child with mental disability, and physical aggression coming from the partner. Regarding violence against the children, the father is the perpetrator of both forms observed in this family.

Genogram F (Figure 1) shows a family arrangement constituted of father, mother, two children, paternal aunt, and six cousins (children of different partners). The dysfunctional dynamics can be observed by the lack of bonds established between the paternal aunt and her children. Maternal bonding is deteriorated by chemical dependence (alcoholism), conflicting affectional relationship, and facilitation of sexual abuse (perpetrated by an uncle toward the nieces). In this family arrangement, the only male figure (father/maternal uncle) established an abusive bond, as he is the perpetrator of sexual violence against the children (nieces).

Figure 2 (genogram S) illustrates an extended family arrangement, formed by three generations living in the same household: father, mother, and children, grandmother (head of the family) and paternal sister, husband, and child. Parental bonding is characterized by persistent physical and emotional aggression caused by the parents. In the maternal bond, degradation is stronger due to the mental condition of the mother, and there is a relationship of physical abuse towards the younger children. The father, who is chemically dependent, established a poor bond with the younger children; with the older one, the relationship involves emotional abuse. It is possible to observe repetition of the chemical dependence behavior in a direct line of parentage, passing from the paternal sister to the adolescent nephew. Regarding violence against the children, in this family arrangement the perpetrators were the parents.

Genogram R (Figure 2) presents a nuclear family arrangement (father, mother, and children); however, the quality of parental bonding is extremely degrading. There is presence of multiple drug abuse by both parents. The paternal connection with the child is distant, and the emotional relationship involves physical abuse. The mother has a connection characterized by intense and violent fusion. In this family arrangement, the child depends exclusively on the mother, therefore being submitted to a large spectrum of violence (negligence, physical, and emotional aggression).

## DISCUSSION

The families assisted at the daycare facility, living in a condition of social vulnerability, represent a heterogeneous community, concerning both family arrangements and the quality of parental bonding. When the pediatrician evaluates the child guided by the practice that is limited to the biomedical model, it is not possible to consider the complexity of interactions in the family household, which contribute to the child’s health. The action of not recognizing family dynamics may result in limited and mistaken interventions addressed to determining the real needs of the patient and his or her family, which is negative for full care.

Therefore, the use of the family genogram will enable the analysis of the psychosocial context of the children, their families and diseases, favoring the identification of stressful factors in the family context. Its applicability starts in the pediatric outpatient clinic, contributing to the construction of clinical reasoning and judgment, and goes to emergency care situations, in which the traditional model does not provide satisfactory answers to the clinical situations of the child. The conditions in which the biomedical model shows its limitations the most involve complex family contexts, with children’s behavioral issues, mental disorder, chemical dependence, and intra-household violence.^13^ ^,^ ^14^

The children in this study did not have clinical complaints; however, by analyzing their genograms, the vulnerability to several forms of violence became clear. All families assessed, showed high level of social vulnerability, including different family arrangements and a wide spectrum of parental bonding quality, reaching extreme degradation. In these families, the bond is weakened by maternal mental disorders, single-parent relationships with the absence of the father, chemical dependence or presence of multiple partners. It was observed that violence defined as the intentional use of physical force or power, actually put to use or threatened, self-inflicted, inter-personal or collective, leading to or with high chances of injury, psychological damage, developmental deficiencies, or privation and death, which were the reality of these families - with a variation in the level of intense and type.^15^ Violence between parents teaches children lessons of power, and turns them into aggressors in the future. Children coming from violent homes are more aggressive with their peers, more similar to the aggressor, and also victims. There is a high correlation between the abuse of these children and violent homes.^16^ All genograms presented in this study showed different forms of violence, ranging from verbal, physical violence, and negligence to sexual abuse. In some family arrangements, more than one type of violence was practiced against the same child. In this sample, all family groups presented violence and the presence of some sort of abuse against children. Physical violence against children is present in the families of M, S, and R. Sexual abuse against children is observed in M and F’s families.

In dysfunctional family arrangements, the child’s physical and psychic health is under constant threat. Therefore, if the children and their families do not receive the proper assistance from supportive and protective networks, they can evolve to a long cycle of disturbances, thus perpetuating violence.^17^ The use of the genogram as a tool that is able to identify the degradation of parental bonding and violence against children has proven to be effective in order to diagnose the need of full and multi-professional care of these families. Based on this diagnosis, it is possible to elaborate a family therapy project comprehending sensitive issues, such as chemical dependence and sexuality. With this type of intervention, it is possible to interrupt the repetition of transgenerational behavior,^18^ as observed in genogram S, in which the mother’s chemical dependence is reproduced by her teenage son.

The life of this child with alcoholic parents has been associated with the development of mental and behavioral disorders, such as depression, anxiety, affectional disorders, low school performance, and emotional insecurity.^19^ ^,^ ^20^ ^,^ ^21^ Other studies also pointed out to the association between family violence combined with alcohol or drug abuse, by one or both parents, with the development of dysthymia, conduct disorder, major depression, chemical dependence, and post-traumatic stress syndrome. ^22^ ^,^ ^23^ ^,^ ^24^

In the four families assessed with the genogram, the presence of family violence associated with alcohol or drug abuse by one or both parents was observed; in these cases, the children of these families had their parents as their single affectional bond. Therefore, all children in these families are subject to developing some sort of mental or behavioral disorder throughout their lives. According to the daycare, at least one child in every analyzed family presented difficulties in school performance, in socializing with other kids, and in establishing an affectional bond with the teachers.

The results of this study demonstrate the importance of using the family genogram in the pediatric appointment, in extreme cases, as a tool to analyze the degradation of parental bonding and violence against children; however, this resource also allows to assess the exposure of the child to several types of stressful factors in less extreme cases. The genogram allows the pediatrician to assess the structure and the functionality of the family, as well as the role of the child in the household. Its construction is dynamic and should be completed after each appointment, according to the transformations in the lifecycles of the family. Therefore, the quality of parental bonding is shown, and violence against children can be perceived in its genesis, even at milder levels.

Despite being similar to a genealogical tree, its complexity and comprehension is much larger. Its configuration is more like a relational map of family members, whose symbols have been standardized since the early 1980s, by the North-American Care Research Group. The symbols characterizing the male individuals are squares, and female figures are circles. The affectional relationships are drawn with lines, according to the intensity and type. The graphic presentation in a single page, in the medical record, allows the health team involved in childcare to be aware of the available data. This information often involves delicate situations, regarding difficult family relations, which are, however, relevant to understand an acute or chronic condition and to construct adequate therapeutic projects.

Its incorporation in different scenarios of pediatric clinical practice could, in fact, contribute to the promotion of full health care for the child, increase the resoluteness of the care provided, assist the family to find new ways to see its members, promote more ways to rescue autonomy in their own therapeutic plan regardless of the presence of social vulnerabilities.
